# What Do They Want from Us? A Survey of EM Program Directors on EM Application Criteria

**DOI:** 10.5811/westjem.2016.10.31496

**Published:** 2016-11-23

**Authors:** Kevin King, Dara Kass

**Affiliations:** * The University of Texas Health Science Center at San Antonio, Department of Emergency Medicine, San Antonio, Texas; † New York University School of Medicine, Department of Emergency Medicine, New York, New York

## Abstract

**Introduction:**

Although a relatively young specialty, emergency medicine (EM) is popular among medical students and is one of the most competitive large specialties. Consequently, students increasingly seek more opportunity to differentiate themselves from their colleagues by pursuing more clerkships at the cost of taking out additional loans: this despite the fact that those who match in EM typically do so in their top three choices. We sought to ascertain what factors EM program directors seek in their typical candidate.

**Methods:**

We recruited EM program directors via the Council of Emergency Medicine Residency Directors email listserv to participate in an anonymous survey regarding the United States Medical Licensing Examination (USMLE), the number of standardized letters of evaluation (SLOE), and the number of EM rotations during the fourth year.

**Results:**

135 respondents completed the anonymous survey: 59% of respondents stated their program did not have a minimum USMLE Step 1 score, but 39% reported a minimum score of 210 or higher; 95% of programs do not require Step 2 to grant an interview, but 46% require it to place the student on the rank list; 80% require only one EM rotation to grant an interview and none require more than two; 95% of programs will accept two SLOEs for both application and rank list placement.

**Conclusion:**

For the typical EM applicant, there is likely little benefit to performing more than two rotations and obtaining more than two SLOEs. Students can defer USMLE Step 2 but must complete it by the time rank lists are due. Our study was limited by the anonymity of the survey, and comments by the respondents revealed the questions did not account for some nuances programs apply to their application review process.

## INTRODUCTION

Although emergency medicine (EM) is a young specialty, it is a popular career choice for graduating medical students. Internal medicine, family medicine, pediatrics and EM offer the highest number of categorical residency positions.^1^ Despite consistent increases in the number of training spots, last year there was only one unmatched spot in EM, making it the most competitive of the aforementioned specialties.^1^

The residency match process is stressful for both advisors and medical students. Students applying to competitive specialties, such as EM, are being told to apply to an increasing number of residency programs.^2^ Although students are applying to a record number of programs, 80% of matched candidates ultimately match at one of their top three choices.^1^

Published recommendations for students applying to EM have impacted how we advise students. In 2000, Crane et al surveyed EM program directors (PD) to understand which aspects of the EM application were important to them.^3^ They found that EM rotation grades, residency interview, clinical grades and clinical recommendations were the most important aspects of the EM application. More recent data from the NRMP corroborate that as well.^4^

In 2016, Clerkship Directors in Emergency Medicine (CDEM) published an advising guide for students applying to EM. This guide, written by experienced EM clerkship directors, addresses the question, *“If 3 is good, 4 is better, right??”*^5^ This referred to the incremental increase in number of away rotations students were pursuing. Despite knowledge that strong EM rotation grades and the standardized letter of evaluation (SLOE) carry significant weight^6^ in an EM residency application, students often buffer their applications with multiple away rotations and apply to dozens of residency programs, irrespective of the overall quality of their candidacy.^5^ While the clerkship directors declared that more is not necessarily better in respect to rotations and SLOEs, we felt that the addition of EM residency PD attitudes would reinforce the message of the CDEM guide.

The increase in the number of away rotations and Electronic Residency Application Service (ERAS) applications comes at a real cost to the student and the system.^2^ Away rotations are a fixed resource and without a centralized mechanism for tracking students and rotations, with some students struggling to secure even one away rotation. The monetary costs of the residency application process causes EM-bound medical students to take out additional loans in their final year and accrue an average of $4,000 in debt to pay for the residency application process.^2^

We set out to understand what EM PDs require during two phases of the application process: granting an interview and placing an applicant on the rank list. We hope that students and their advisors can use this information and that published by CDEM, to formulate an efficient application strategy.

We hoped this information, coupled with the knowledge that the majority of applicants match in one of their top choices, could be used to accurately advise EM-bound medical students, reducing the number of “extra” away rotations and residency applications students seek.

## METHODS

We designed a survey to collect the information from EM residency PDs toward EM residency applications. The survey contained 18 questions pertaining to United States Medical Licensing Examination Step 1 and Step 2, EM rotations and SLOEs in reference to interview offers and rank list placement. (See Addendum 1.)

We created the survey in Google Forms and distributed an invitation to PDs to participate on two dates in December 2015 and January 2016, electronically, via the Council of Emergency Medicine Residency Directors email (CORD) listserv. Responses were collected over the subsequent 30-day period. Respondents were asked to report their SAEM regional location, but their identity and program affiliation were not tracked. We collected data anonymously via Google Forms.

This project was reviewed by the University of Texas Health Science Center Institutional Review Board (IRB) and deemed exempt from informed consent.

## RESULTS

We received 135 responses to the survey. At the time of the survey, there were 198 domestic EM residency programs on the CORD listserve (as per CORD headquarters, personal correspondence). SAEM regional response can be seen in Table.

### USMLE Scores

Four questions referred to USMLE Step 1 and Step 2: 59% of respondents stated they did not have a minimum score to grant an interview and 39% required a minimum score of 210 or higher. While 95% of respondents reported they did not require Step 2 to grant an interview, 46% required it to place an applicant on the rank list.

### EM Rotations

Eighty percent of respondents require at least one EM rotation to grant an interview and 20% required two. No respondents required more than two. Twenty-seven percent (27%) reported that it was “critical” for a student to complete a home rotation, even if they have no intention of attending residency there.

### SLOEs

To grant an interview, 97% of respondents required two or fewer SLOEs and 95% required two or fewer to place an applicant on their rank list. Forty-seven percent (47%) of respondents required one letter and 10% reported requiring no SLOE to grant an interview. Respondents were split on personal letters in lieu of a SLOE, with 46% reporting they would not accept a personal letter.

### Application Timing

The timing of completion of application packets is important. Only 15% of respondents reported that it was “highly likely” that an application would be reviewed if it were completed after the ERAS opening date in September.

## DISCUSSION

The competitive nature of EM residency applications has resulted in applicant anxiety regarding away rotations, application submission and USMLE scores. We asked EM PDs what their minimum criteria were for interview extension and rank list submission.

Most EM programs do not have a minimum Step 1 score to grant an interview. For those that do, cutoff scores falls between 200–220 and many programs note that they “almost always” grant interviews to students with Step 1 score above 230.^4^ It is reasonable to expect that most students with a Step 1 score greater than 230 will meet the screening criteria for most residency programs. USMLE Step 2 was not required by the vast majority of respondent programs for interview extension. However, nearly half required those results to place an applicant on the program rank list. Therefore, students with adequate Step 1 scores should be able to defer taking Step 2 until sometime in the late fall to permit more flexibility in scheduling rotations. Finally, all students should try to have USMLE Step 2 results in ERAS by the time programs have finalized their rank lists for submission.

Regarding EM rotations, students may want to rotate at their home institution, regardless of their interest in matching at that program, since one-quarter of respondents wanted to see a SLOE from the “home institution.” Our data support that most students only need to complete two rotations unless their advisor feels an additional rotation is needed to strengthen their application.

## LIMITATIONS

This study was designed to provide initial, pilot data to assist the CDEM Student Advisory Task Force in developing student-advising guidelines for the EM residency application process.

As a pilot study, it has several important limitations. Anonymous participants were recruited via the CORD listserv and multiple responses from the same individual or the same residency program were possible. Yet our regional response data suggest a wide variety of programs participated in the survey.

We did allow for free-text comments on our survey, which may have affected how people responded to specific questions. However, we did not perform an analysis on the responses due to time and resource constraints. Additionally, due to incomplete or conflicting data and responses, some survey questions were excluded from this analysis.

The survey itself was not a validated instrument and was intended only as a barometer of overall opinion of residency programs. It is inadvisable to draw specific conclusions about a specific applicant from these data and advisors should incorporate this information into the overall advising they provide to their students.

## CONCLUSION

The anxiety of students and their advisors has resulted in an increase of EM applications and away rotations with an accompanying increased cost for both the students and medical schools. By using multiple sources of information such as the National Residency Matching Program data, this survey and other sources, advisors can reduce the cost and complexity of student EM residency application while providing a reasonable expectation of a successful match. While our small preliminary study provides insight into the attitudes of EM residency program directors regarding residency applications, it falls short of comprehensive recommendations or guidelines. The further development of this area of study would undoubtedly assist students, residency programs and medical schools to develop rational, cost-effective application strategies.

## Figures and Tables

**Table:** Emergency medicine program directors’ response to survey on applicant criteria, by SAEM region.

SAEM region	% Respondents (n=135)
Great Plains	5.6% (7)
New England	9.6% (12)
Mid-Atlantic	18.4% (23)
Midwest	25.6% (32)
Southeastern	22.4% (28)

*SAEM*, Society of Academic Emergency Medicine.
